# Integrated DNA/RNA targeted genomic profiling of diffuse large B-cell lymphoma using a clinical assay

**DOI:** 10.1038/s41408-018-0089-0

**Published:** 2018-06-12

**Authors:** Andrew M. Intlekofer, Erel Joffe, Connie L. Batlevi, Patrick Hilden, Jie He, Venkatraman E. Seshan, Andrew D. Zelenetz, M. Lia Palomba, Craig H. Moskowitz, Carol Portlock, David J. Straus, Ariela Noy, Steven M. Horwitz, John F. Gerecitano, Alison Moskowitz, Paul Hamlin, Matthew J Matasar, Anita Kumar, Marcel R. van den Brink, Kristina M. Knapp, Janine D. Pichardo, Michelle K. Nahas, Sally E. Trabucco, Tariq Mughal, Amanda R. Copeland, Elli Papaemmanuil, Mathai Moarii, Ross L. Levine, Ahmet Dogan, Vincent A. Miller, Anas Younes

**Affiliations:** 1 Lymphoma Service, Department of Medicine, Memorial Sloan Kettering Cancer Center, New York, NY USA; 2 Department of Medicine, Weill Cornell Medical College, New York, NY USA; 3 Human Oncology and Pathogenesis Program, Memorial Sloan Kettering Cancer Center, New York, NY USA; 4 Center for Hematologic Malignancies, Memorial Sloan Kettering Cancer Center, New York, NY USA; 5 Department of Epidemiology and Biostatistics, Memorial Sloan Kettering Cancer Center, New York, NY USA; 6 Foundation Medicine Inc., Cambridge, MA USA; 7 Division of Hematologic Oncology, Memorial Sloan Kettering Cancer Center, New York, NY USA; 8 Department of Pathology, Memorial Sloan Kettering Cancer Center, New York, NY USA; 9 Leukemia Service, Department of Medicine, Memorial Sloan Kettering Cancer Center, New York, NY USA

## Abstract

We sought to define the genomic landscape of diffuse large B-cell lymphoma (DLBCL) by using formalin-fixed paraffin-embedded (FFPE) biopsy specimens. We used targeted sequencing of genes altered in hematologic malignancies, including DNA coding sequence for 405 genes, noncoding sequence for 31 genes, and RNA coding sequence for 265 genes (FoundationOne-Heme). Short variants, rearrangements, and copy number alterations were determined. We studied 198 samples (114 de novo, 58 previously treated, and 26 large-cell transformation from follicular lymphoma). Median number of GAs per case was 6, with 97% of patients harboring at least one alteration. Recurrent GAs were detected in genes with established roles in DLBCL pathogenesis (e.g. *MYD88*, *CREBBP*, *CD79B*, *EZH2*), as well as notable differences compared to prior studies such as inactivating mutations in *TET2* (5%). Less common GAs identified potential targets for approved or investigational therapies, including *BRAF*, *CD274* (PD-L1), *IDH2*, and *JAK1/2*. *TP53* mutations were more frequently observed in relapsed/refractory DLBCL, and predicted for lack of response to first-line chemotherapy, identifying a subset of patients that could be prioritized for novel therapies. Overall, 90% (*n* = 169) of the patients harbored a GA which could be explored for therapeutic intervention, with 54% (*n* = 107) harboring more than one putative target.

## Key points

- This study demonstrates the applicability of CLIA-compliant targeted FFPE sequencing for large-scale clinical trials.
- *TP53mut* is the main predictor of refractoriness or early relapse.

## Introduction

The ability to identify and therapeutically target patient-specific genomic alterations has made precision oncology a reality for several types of cancer^1^. Unfortunately, in aggressive lymphomas, no approved genomic biomarker-driven therapies are standard of care. The problem is exemplified by diffuse large B-cell lymphoma (DLBCL) where, despite a relapse rate of over 30%, RCHOP is being administered as an almost uniform first-line of care, over two decades since it was introduced^2^. Thus, there is an unmet need to develop genomic biomarker-driven therapeutics, to improve outcomes for patients with DLBCL.

Next-generation sequencing studies have produced a vast array of data regarding the underlying genomic alterations (GAs) that characterize DLBCL. These demonstrate a striking genetic heterogeneity that likely accounts for the observed variability in clinical phenotype^3–7^. Recurrent alterations have been identified in over 300 genes, none of which pathognomonic, as all occur at frequencies <30%, usually <10%^7^. Identifying the clinical implications of these alterations requires large cohorts, and the integration of several testing modalities (e.g. DNA sequencing to identify short nucleotide variations (SNVs) and copy number alterations (CNAs), and RNA sequencing for gene rearrangements)^8^. In this regard, most genomic studies in DLBCL have been carried out in the research setting, often implementing assays such as whole genome or whole exome sequencing using fresh frozen biopsy specimens^7^. These may be prohibitively resource intensive for adaptation in large-scale clinical trials or in everyday practice. In these settings, integrated hybridization capture of both DNA and RNA using formalin-fixed paraffin-embedded (FFPE) specimens may be most appropriate^7,9^. Potential advantages of this approach include: (1) simultaneous detection of all classes of GAs including short variants (base substitutions and small indels), CNAs, and gene rearrangements/fusions; (2) sensitive detection of fusion transcripts involving the genes of interest due to inclusion of the RNA sequencing component; (3) flexibility in sample acquisition, storage and transportation when using FFPE; (4) high depth of sequencing coverage to enhance detection of rare variants even in samples with extensive non-malignant stromal and immune cell contamination; (5) streamlined bioinformatics; and (6) compliance with The Clinical Laboratory Improvement Amendments (CLIA) standard. However, it remains to be established what spectrum of GAs will be observed in clinical FFPE DLBCL specimens, how specific GAs will correlate with clinical and pathologic phenotype and with patient outcomes, and how this information can be incorporated in clinical care.

Herein, we describe the application of commercially available, CLIA-compliant, integrated DNA and RNA targeted sequencing panel (FoundationOne-Heme) to a retrospective cohort of 198 FFPE DLBCL specimens for the identification of GAs with potential clinical significance.

## Materials and methods

### Study population

Archived FFPE biopsy specimens from 198 patients with DLBCL were obtained with approval from the Memorial Sloan Kettering Cancer Center (MSKCC) Institutional Review Board. Biopsies were collected between 1989 and 2012. Inclusion criteria were histologically confirmed DLBCL with appropriate patient consent to perform genomic sequencing. Germinal center B-cell-like (GCB) or non-GCB cell-of-origin (COO) was assessed by immunohistochemistry (IHC) according to the Hans algorithm^8^. Baseline demographics and survival data were extracted from the clinical record.

### Sample preparation and sequencing

Samples were sequenced using the FoundationOne-Heme platform that uses DNA sequencing to interrogate the entire coding sequence of 406 genes, selected introns of 31 genes involved in rearrangements, and utilizes RNA sequencing to interrogate 265 genes known to be somatically altered in human hematologic malignancies^10^. Detailed protocols for DNA and RNA extraction, cDNA synthesis, library construction, and hybrid selection as well as a survey of methodological validation tests, have been recently published, and are detailed in the supplementary methods and supplementary table 1 ^10,11^. In brief, specimens were reviewed by a pathologist to confirm ≥20% tumor nuclei and a tissue volume of ≥2 mm^3^. We used 20% tumor content as the minimal requirement, having demonstrated in a previous validation study that the pipeline approaches a sensitivity of 100% for SNVs and CNAs above this cutoff^10^. Genomic DNA and RNA were extracted and fragmented to ~200 bp fragment size. Samples were tested to ensure sufficient DNA yield (50–200 ng) and RNA yield (≥3.5 ng/µL). Sequencing was performed with the Illumina HiSeq2500 system using 49 × 49 paired-end reads. Resultant sequences were analyzed for single nucleotide variants (SNVs—base substitutions and small indels), CNAs and rearrangements. Samples with median coverage <150× were considered failed and excluded from analysis. Known germline variants (per 1000 Genomes Project) were removed^10,11^. Significant non-synonymous variants were defined as any somatic alteration annotated in the COSMIC database (v62), as well as clear inactivating mutations (i.e. truncations or deletions) in established tumor suppressor genes^10,11^. The mutant allele frequency cutoff used for known somatic variants was 1%; 5% for potential driver somatic variants; 3% for previously described indels; and 10% for potential driver indels. Gene amplifications/gains were defined at a copy number ≥6, and gene losses as copy number of 0 ^10,11^. For rearrangement identification we required a minimum of ten chimera reads for known fusions and 50 for potential driver rearrangements. Any aberration not meeting the aforementioned criteria was defined as *Unknown Significance* (UKS). Sequencing data are publicly available in an interactive format through the cBioPortal (www.cbioportal.org).

Individual genes were grouped together by the biologic pathways in which they operate (supplementary table 2)^12,13^. Actionable variants and pathways were defined by the presence of GAs predictive of response to an FDA-approved drug or an experimental agent in clinical trial^14^. These data were derived by a review of literature and publicly accessible databases including OncoKB, the FDA Pharmacogenomic Biomarkers in Drug Labeling, GeneCards, and clinicaltrials.gov^12–17^. Variant actionability was graded based on the OncoKB criteria. Level 1 was defined as alterations recognized by the FDA as predictive of response to an approved drug in DLBCL. Level 2 included non-FDA predictive biomarkers for response in DLBCL (2A) or FDA-approved biomarkers for response in a different malignancy (2B). Level 3 includes alterations supported by compelling data from clinical trials in DLBCL (3A) or another malignancy (3B). Level 4 are candidate biomarkers for response based on early clinical or preclinical studies (supplementary excel file 2)^14^.

### Statistical analysis

Descriptive statistics are provided for all genes dichotomously (i.e. presence/absence of any alteration). Differences in alteration frequency between groups were determined using Fischer’s exact test, with differences in the total number of alterations across groups assessed using a Wilcoxon rank-sum test. Clustering analysis was done based on the Jaccard distance using the Ward D method. Analyses for response to treatment and survival were performed in the subset of patients with de novo disease treated with RCHOP (rituximab, cyclophosphamide, adriamycin, vincristine, steroids) or RCHOP-like chemotherapy. For the purpose of these analyses, tFL not previously treated was included with the de novo group, reasoning that in clinical practice the distinction between tFL at first diagnosis and DLBCL is not made easily, such that both conditions are treated similarly as de novo DLBCL and have comparable outcomes^18^. Median follow-up was estimated using the reverse Kaplan−Meier method. Overall and progression-free survival (OS/PFS) were defined as the time from initiation of frontline treatment until death of any cause or disease progression or death (for PFS), censoring at the end of follow-up. Differences in OS and PFS between groups were assessed using the Kaplan−Meier method as well as univariate Cox proportional hazards regression models. Where applicable we adjusted for false discovery (FDR) using the Benjamini−Hochberg approach. Analyses were done in R 3.4.0 (R foundation, Austria).

## Results

Of 219 FFPE DLBCL samples attempted, 214 were successfully sequenced, indicating a success rate of 98%. Sixteen cases were excluded from the analysis (for inadequate clinical data, primary central nervous system lymphoma or large-cell transformation from indolent lymphomas other than FL), leaving 198 cases for this analysis: 114 cases were from newly diagnosed untreated patients (de novo), 58 from previously treated patients, and 26 from tFL cases. Cell of origin was determined in 177 cases, with 48% (*n* = 95) classified as GCB and 41% (*n* = 82) as non-GCB. Of the 114 patients sequenced at diagnosis, 30% (*n* = 35) were refractory to first-line treatment or subsequently relapsed during follow-up. The median unique sequencing coverage was 555 × [476–656] for DNA and median total pairs for RNA were >20×10^6^.

### Genomic alterations and pathways landscape

**Fig. 1:**
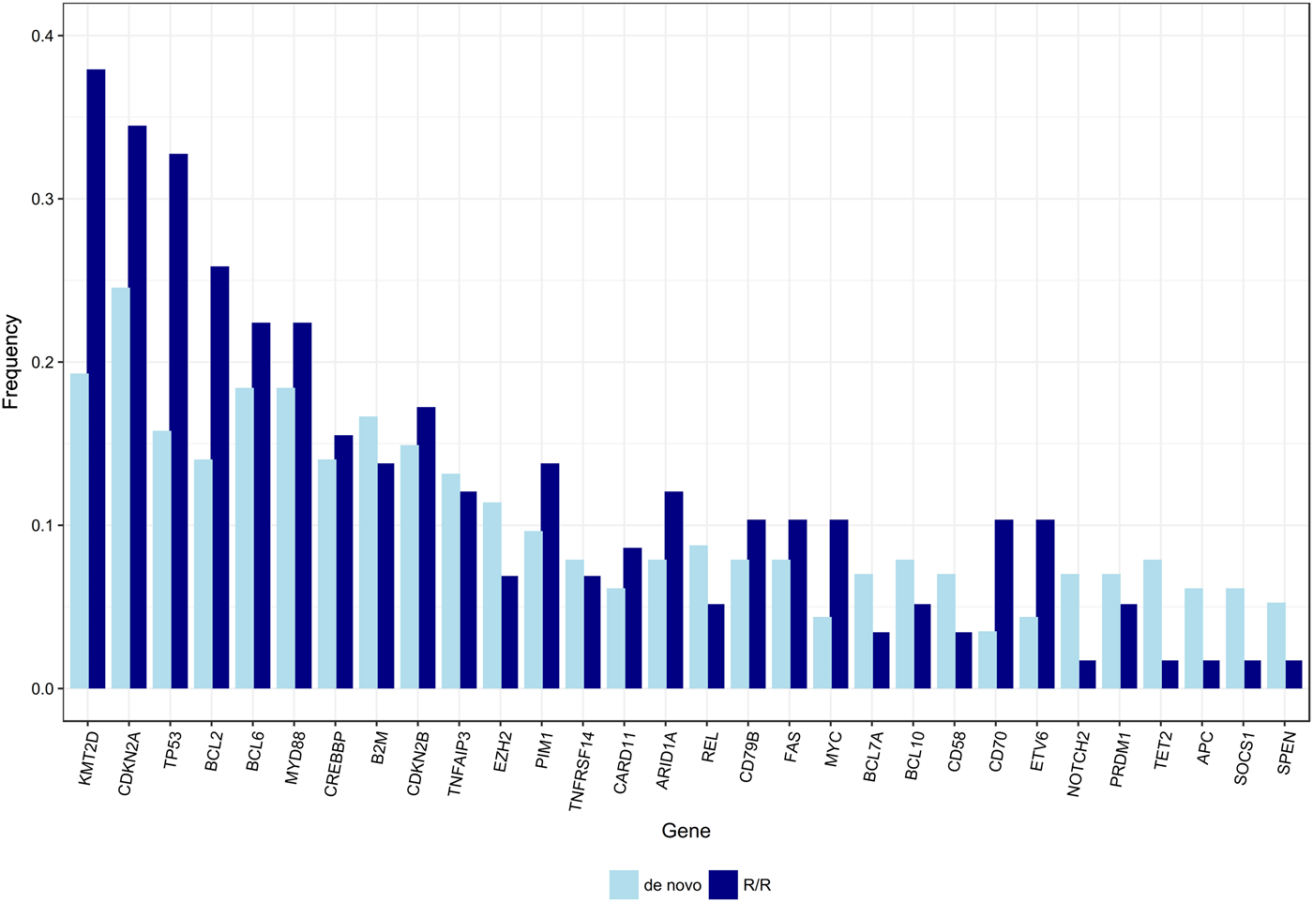
Genomic alterations in de novo vs. R/R disease. Bar plot of genomic alterations present in ≥5% of the subjects by order of frequency and by R/R status. tFL cases are not presented. The significantly different GAs were *TP53mut* (*p* = 0.02) and *KMT2Dmut* (*p* = 0.01)

**Fig. 2:**
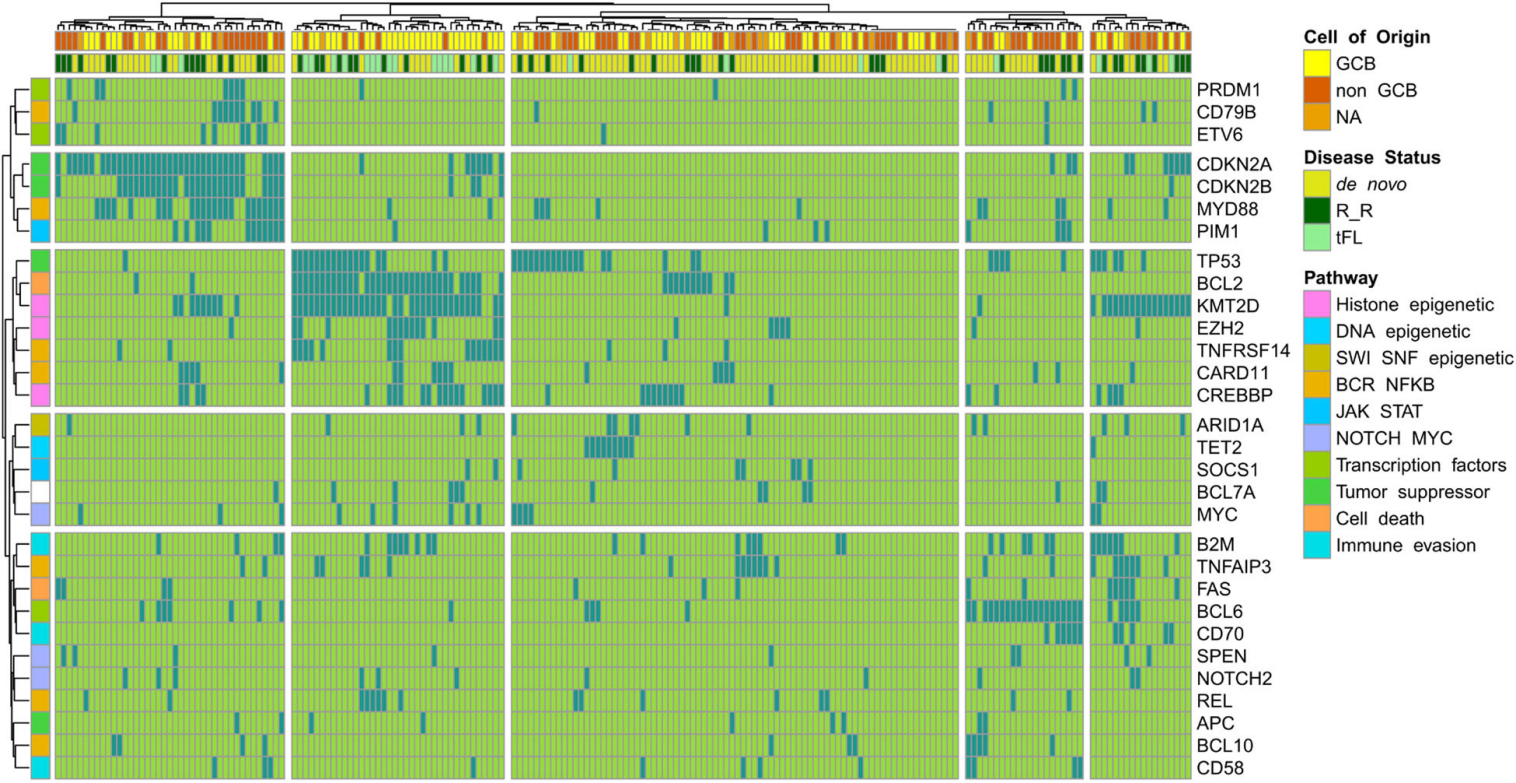
Genomic alteration clusters annotated by cell of origin and molecular pathway. Cluster analysis based on co-occurrence/anti-co-occurrence distance (“Jaccard”) with annotated pathways, R/R status (irrespective of time of sequencing) and cell of origin (by IHC). Presented are only genomic abnormalities present in ≥5% of the cohort. Dark-green/light-green (main plot)—presence/absence of a genomic abnormality respectively

The median number of GAs per case was 6, with 97% of patients harboring at least one alteration (Table 1, supplementary figure 1 and supplementary excel file 1). The most commonly identified SNVs were in *KMT2D* (MLL2; 31%, *n* = 62), *TP53* (24%, *n* = 48), *MYD88* (18%, *n* = 36), *CREBBP* (18%, *n* = 35), and *B2M* (Beta-2-microglobulin; 17%; *n* = 33) (Fig. 1, Table 1). CNAs were identified in 42% (*n* = 84) of cases, involving 37 different genes. The most frequently identified losses were *CDKN2A* and/or *CDKN2B* (20% combined, *n* = 40), while the most frequent gene amplifications were observed in *REL* (8%, *n* = 16), *CD274* (3%; *n* = 6), and *MCL1* (3%, *n* = 6). Rearrangements (*trans*) were detected in 57% (*n* = 112) of cases involving 61 different genes. As expected, most involved the translocation of *BCL2*, *BCL6*, or *MYC* to the immunoglobulin heavy chain (*IGH*) enhancer (supplementary table 3). Deletion of *CDKN2B* was always accompanied by deletion of *CDKN2A* (though not vice versa) and associated with *CD79Bmut*, *MYD88mut*, *PIM1mut*, and *PRDM1mut* (Fig. 2, supplementary figure 2). *CDKN2Bdel* and *CDKN2Adel* were mutually exclusive with *TP53mut* (*p* < 0.001) as were *BCL10mut* (*p* = 0.04) and *CD58mut* (*p* = 0.04). A cluster of *BCL2trans* and *KMT2Dmut* corresponded with a GCB subtype and with high rates of *TP53mut*, *EZH2mut*, and *TNFRSF14mut* (*p* = 0.002; Fig. 2). Of note, the largest cluster of 80 patients (40%) did not have a distinct genomic signature.

**Table 1:** Summary of key genomic alterations by disease status at time of sequencing

	[ALL]	de novo	R/R	tFL	*p* value*	BH p*
	198	114	58	26		
Cell of origin						
GCB	95 (48.0%)	51 (44.7%)	26 (44.8%)	18 (69.2%)	0.602	
Non-GCB	82 (41.4%)	51 (44.7%)	23 (39.7%)	8 (30.8%)		
NA	21 (10.6%)	12 (10.5%)	9 (15.5%)	0 (0.00%)		
SNVs	191 (96.5)	108 (94.7)	57 (98.3)	26 (100.0)	0.426	
SNVs per/pt. (min, max)	4 (0, 9)	4 (0, 9)	4 (0, 9)	4.5 (1, 9)	0.640	
SNVs of UKS per/pt.	15.5 (3, 45)	16 (3, 45)	15.5 (4, 28)	13.5 (6, 23)	0.894	
Amplifications	36 (18.2)	20 (17.5)	10 (17.2)	6 (23.1)	1.000	
Deletions	57 (28.8)	28 (24.6)	20 (34.5)	9 (34.6)	0.233	
Translocations	**112 (56.6)**	**51 (44.7)**	**41 (70.7)**	**20 (76.9)**	**0.002**	
Total number of GAs	6 (0, 13)	5 (0, 13)	6 (0, 13)	7 (1, 11)	0.078	
KMT2D	**62 (31.3%)**	**22 (19.3%)**	**22 (37.9%)**	**18 (69.2%)**	**0.014**	**0.253**
CDKN2A	54 (27.3%)	28 (24.6%)	20 (34.5%)	6 (23.1%)	0.233	0.726
TP53	**48 (24.2%)**	**18 (15.8%)**	**19 (32.8%)**	**11 (42.3%)**	**0.018**	**0.253**
BCL2	46 (23.2%)	16 (14.0%)	15 (25.9%)	**15 (57.7%)**	0.090	0.501
BCL6	37 (18.7%)	21 (18.4%)	13 (22.4%)	3 (11.5%)	0.675	0.934
MYD88	36 (18.2%)	21 (18.4%)	13 (22.4%)	2 (7.69%)	0.675	0.934
CREBBP	35 (17.7%)	16 (14.0%)	9 (15.5%)	10 (38.5%)	0.975	1.000
B2M	33 (16.7%)	19 (16.7%)	8 (13.8%)	6 (23.1%)	0.789	0.934
CDKN2B	32 (16.2%)	17 (14.9%)	10 (17.2%)	5 (19.2%)	0.861	0.964
TNFAIP3	24 (12.1%)	15 (13.2%)	7 (12.1%)	2 (7.69%)	1.000	1.000
EZH2	21 (10.6%)	13 (11.4%)	4 (6.90%)	4 (15.4%)	0.505	0.934
PIM1	20 (10.1%)	11 (9.65%)	8 (13.8%)	1 (3.85%)	0.574	0.934
TNFRSF14	20 (10.1%)	9 (7.89%)	4 (6.90%)	7 (26.9%)	1.000	1.000
CARD11	19 (9.60%)	7 (6.14%)	5 (8.62%)	7 (26.9%)	0.541	0.934
ARID1A	16 (8.08%)	9 (7.89%)	7 (12.1%)	0 (0.00%)	0.540	0.934
REL	16 (8.08%)	10 (8.77%)	3 (5.17%)	3 (11.5%)	0.547	0.934
CD79B	15 (7.58%)	9 (7.89%)	6 (10.3%)	0 (0.00%)	0.801	0.934
FAS	15 (7.58%)	9 (7.89%)	6 (10.3%)	0 (0.00%)	0.801	0.934
MYC	15 (7.58%)	5 (4.39%)	6 (10.3%)	4 (15.4%)	0.186	0.650
BCL7A	14 (7.07%)	8 (7.02%)	2 (3.45%)	4 (15.4%)	0.498	0.934
BCL10	12 (6.06%)	9 (7.89%)	3 (5.17%)	0 (0.00%)	0.753	0.934
CD58	12 (6.06%)	8 (7.02%)	2 (3.45%)	2 (7.69%)	0.498	0.934
CD70	11 (5.56%)	4 (3.51%)	6 (10.3%)	1 (3.85%)	0.089	0.501
ETV6	11 (5.56%)	5 (4.39%)	6 (10.3%)	0 (0.00%)	0.186	0.650
NOTCH2	11 (5.56%)	8 (7.02%)	1 (1.72%)	2 (7.69%)	0.276	0.772
PRDM1	11 (5.56%)	8 (7.02%)	3 (5.17%)	0 (0.00%)	0.752	0.934
TET2	10 (5.05%)	9 (7.89%)	1 (1.72%)	0 (0.00%)	0.167	0.650

Only alterations observed within at least ten patients are included

Differing values with unadjusted *p* < 0.05 depicted in bold

*BH* FDR-adjusted *p* value (Benjamini−Hochberg), *R/R* relapsed refractory, *NA* not available, *SNV* short nucleotide variant, *tFL* transformed follicular lymphoma

*Unadjusted and BH-adjusted *p* values reflect the comparison of R/R to de novo disease (i.e. excludes tFL)

Patients with R/R disease and tFL had higher rates of *TP53mut* compared to de novo patients (16% *n* = 18 de novo, 33% *n* = 19 R/R; and 42% *n* = 11 tFL; *p* = 0.02 R/R vs. de novo), and of *KMT2Dmut* (19% *n* = 22 de novo, 38% *n* = 22 R/R; and 69% *n* = 18 tFL; *p* = 0.01 R/R vs. de novo) (Table 1). Further, R/R and tFL cases were enriched for translocations (45% *n* = 51 de novo, 71% *n* = 41 R/R; and 77% *n* = 20 tFL; *p* = 0.002 for R/R vs. de novo). These were mainly *IGH:BCL2* rearrangements for tFL (75% of cases), while among R/R patients 37% (*n* = 15) had a *BCL2trans*, 27% (*n* = 11) had a *BCL6trans*, and 12% (*n* = 5) had a *MYCtrans*.

**Fig. 3:**
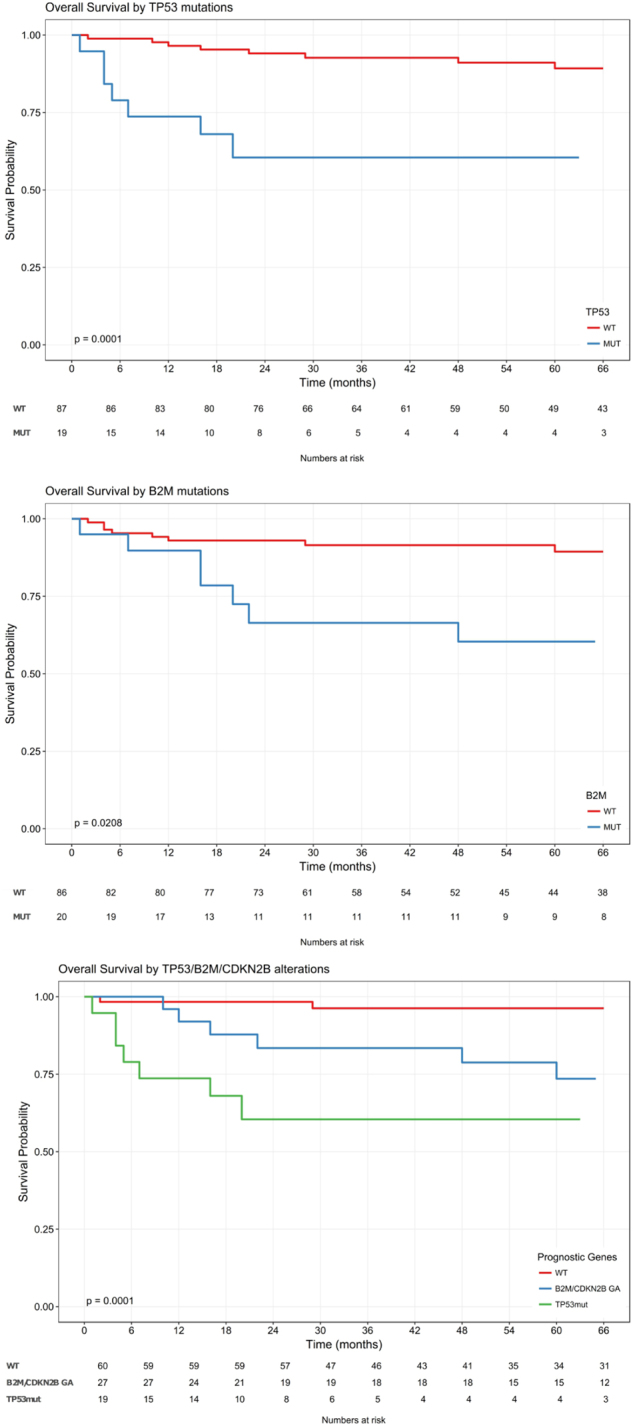
Overall survival by *TP53mut*, *B2Mmut*, and *CDKN2Bdel*

These observations corresponded to a trend towards higher overall rates of abnormalities in tumor suppressors pathways that includes *TP53mut* and *CDKN2Bdel* (54% *n* = 61 de novo, 76% *n* = 44 R/R, and 69% *n* = 18 tFL, *p* = 0.007) and in the epigenetic histone modification pathway which includes *KMT2Dmut* (47% *n* = 41 non-relapsing, 64% *n* = 37 R/R, and 89% *n* = 23 tFL, *p* = 0.008) (Table 2). Of note, after correction for false discovery, none of these differences remained statistically significant. As expected, *BCL2trans* were more common in GCB compared to non-GCB (40% *n* = 38 vs. 5% *n* = 4, *p* < 0.001) as were *CREBBPmut* (27% *n* = 26 vs. 6% *n* = 5, *p* < 0.001), *KMT2Dmut* (43% *n* = 41 vs. 21% *n* = 17, *p* = 0.003), and *TNFRSF14* (17% *n* = 16 vs. 4% *n* = 3, *p* = 0.01). *CD79Bmut* were observed solely in non-GCB (0 vs. 16% *n* = 13, *p* < 0.001), as was an enrichment for *BCL6trans* (11% *n* = 10 vs. 27% *n* = 22, *p* = 0.01) (supplementary table 4 and Fig. 3). We further observed an enrichment in *MYD88mut*, *ETV6mut,* and *PRDM1mut* among non-GCB and *EZH2mut* among GCB tumors; however, these did not remain significant after correction for FDR (supplementary table 5).

**Table 2:** Summary of key involved pathways by disease status at time of sequencing

	[ALL]	de novo	R/R	tFL	*p* value	BH p*
	198	114	58	26		
Tumor suppression	**123 (62.1%)**	**61 (53.5%)**	**44 (75.9%)**	**18 (69.2%)**	**0.007**	**0.079**
Histone epigenetic	**107 (54.0%)**	**47 (41.2%)**	**37 (63.8%)**	**23 (88.5%)**	**0.008**	**0.079**
BCR NFKB	107 (54.0%)	60 (52.6%)	30 (51.7%)	17 (65.4%)	1.000	1.000
Transcription factors	74 (37.4%)	44 (38.6%)	23 (39.7%)	7 (26.9%)	1.000	1.000
Cell death	**68 (34.3%)**	**27 (23.7%)**	**24 (41.4%)**	**17 (65.4%)**	**0.026**	**0.165**
Immune evasion	53 (26.8%)	30 (26.3%)	14 (24.1%)	9 (34.6%)	0.901	1.000
NOTCH MYC	45 (22.7%)	22 (19.3%)	14 (24.1%)	9 (34.6%)	0.590	0.991
JAK STAT	35 (17.7%)	22 (19.3%)	11 (19.0%)	2 (7.7%)	1.000	1.000
RAS MAPK	35 (17.7%)	21 (18.4%)	8 (13.8%)	6 (23.1%)	0.582	0.991
Metabolism	32 (16.2%)	18 (15.8%)	7 (12.1%)	7 (26.9%)	0.670	0.991
Cell cycle	24 (12.1%)	15 (13.2%)	5 (8.6%)	4 (15.4%)	0.531	0.991
Translation	22 (11.1%)	9 (7.9%)	9 (15.5%)	4 (15.4%)	0.200	0.635
SWI SNF epigenetic	21 (10.6%)	12 (10.5%)	9 (15.5%)	0 (0.0%)	0.485	1.000
DNA damage	21 (10.6%)	13 (11.4%)	6 (10.3%)	2 (7.7%)	1.000	0.991
RNA processing	20 (10.1%)	14 (12.3%)	5 (8.6%)	1 (3.8%)	0.641	0.991
Epigenetic cofactors	19 (9.6%)	8 (7.0%)	10 (17.2%)	1 (3.8%)	0.071	0.269
PI3K AKT TOR	17 (8.6%)	10 (8.8%)	7 (12.1%)	0 (0.0%)	0.678	0.991
DNA epigenetic	13 (6.6%)	11 (9.6%)	1 (1.7%)	1 (3.8%)	0.062	0.269
Adhesion cytoskeleton	12 (6.1%)	9 (7.9%)	3 (5.2%)	0 (0.0%)	0.753	1.000

Only alterations observed within at least ten patients are included

Differing values with unadjusted *p* < 0.05 depicted in bold

*R/R* relapsed refractory, *SNV* short nucleotide variant, *tFL* transformed follicular lymphoma

*p values reflect the comparison of R/R to de novo disease (i.e. excludes tFL).

The median number of involved pathways was 4 with 90% of patients having at least two affected pathways (Table 2, Fig. 2). Overall, 90% (*n* = 179) of the patients harbored a GA which could be explored for therapeutic intervention, with 54% (*n* = 107) harboring more than one potential target. GAs involving a gene that is targeted by a drug approved by the FDA for another indication (level 2B) were identified in 56% (*n* = 110). These were comprised mainly of BCL2 inhibitors for *BCL2*-associated GAs, BTK inhibitors for *MYD88mut*, BRAF inhibitors for *BRAFmut* and immune check-point inhibitors in *CD274* and *PDCD1LG2* GAs (supplementary excel file 2). In 41% (*n* = 81) there was a GA targeted by a non-FDA-approved drug with compelling clinical evidence either in DLBCL (level 3A; 33%, *n* = 66; mostly histone deacetylase and EZH2 inhibitors in *CREBBPmut*, *EP300mut*, and *EZH2mut*) or in another indication (level 3B; 8%, *n* = 15). Finally, in 85% there was at least one target for a drug in preclinical or early clinical development (level 4).

### Association of GAs with clinical presentations and pathologic subtypes

There were 106 de novo DLBCL patients treated with an RCHOP-based chemotherapy (58% RCOHP, 36% RCHOP-ICE, 7% DA-EPOCH-R). Median age was 58 (range 21–84) with a 61% male predominance. The majority (75%) had a stage III−IV disease, and 35% had an intermediate-high to high IPI. Complete response was observed in 88% (PR in 3% and SD/PD in 9%) of the patients and was not associated with stage, IPI, or other demographics (Table 3). Median follow-up was 66 months (95% CI 57–73) with 18 deaths and 26 disease progressions documented during that time, representing a 5y OS of 84% (95%CI 77–92%).

**Table 3:** Baseline characteristic of de novo RCHOP/RCHOP-like treated patients by CR attainment

	[ALL]	CR	PR/SD/PD	*p* value
	106*	93	12	
Age (min, max)	58 (21, 84)	58 (21, 83)	51 (31, 84)	0.774
Age > 65	28 (26.4%)	25 (26.9%)	3 (25.0%)	>0.999
Sex (M)	65 (61.3%)	56 (60.2%)	8 (66.7%)	0.907
Disease status				0.320
de novo	96 (90.6%)	85 (91.4%)	10 (83.3%)	
tFL (first presentation)	10 (9.4%)	8 (8.6%)	2 (16.7%)	
Stage				>0.999
I/II	27 (25.5%)	24 (25.8%)	3 (25.0%)	
III/IV	79 (74.5%)	69 (74.2%)	9 (75.0%)	
IPI ≥ 3	37 (34.9%)	31 (33.3%)	6 (50.0%)	0.337
Cell of origin				0.815
GCB	54 (50.9%)	48 (51.6%)	5 (41.7%)	
Non-GCB	41 (38.7%)	35 (37.6%)	6 (50.0%)	
Unclassified	11 (10.4%)	10 (10.8%)	1 (8.3%)	
Treatment				**<0.001**
DAEPOCHR	**7 (6.6%)**	**3 (3.2%)**	**4 (33.3%)**	
RCHOP	**61 (57.5%)**	**52 (55.9%)**	**8 (66.7%)**	
RCHOP_ICE	**38 (35.8%)**	**38 (40.9%)**	**0 (0.0%)**	

*One patient died during frontline therapy and did not have a response designation.

*CR/PR* complete/partial response, *SD/PD* stable/progressive disease

We investigated whether specific GAs were associated with patient outcomes, including response to chemotherapy or OS. We found that *TP53* alterations predicted for lack of response to chemotherapy. Of the 12 patients with primary refractory disease, 8 (67%) were *TP53*mut. Of the 19 patients harboring a *TP53mut*, 10 (53%) did not achieve a complete response (CR) or relapsed within the first year, while one patient relapsed after 3 years. Six were without evidence of disease during a 2–5-year-follow-up, and two were still in remission at 12 and 22 months. *TP53mut* was also associated with shorter OS (5yOS 61% vs. 89% HR 5.8 95%CI 2.1–16, *p* = 0.001). Finally, *TP53mut* were detected in 19 R/R patients, of whom 13 (66%) were either refractory to their previous frontline therapy or had relapsed within less than a year.

Two other GAs were marginally associated with survival. *B2Mmut* (19% of patients; OS HR 2.9, 95%CI 1.1–7.6, *p* = 0.03), and *CDKN2Adel* when accompanied by *CDKN2Bdel* but not alone (14% of patients, OS HR 2.5 95%CI 0.9–7.1, *p* = 0.08). (Table 4, Fig. 3). In addition, abnormalities grouped under the protein translation machinery pathway (e.g. *MYC*, *EIF4A2*), present in 9% of patients, were associated with a shorter OS (HR 4.9 95%CI 1.7–13.8, *p* = 0.003), as were abnormalities in tumor suppressor pathways (which includes *TP53mut*). Lastly, patients with no alteration in *TP53*, *CDKN2B* or *B2M*, had a remarkably long OS (5yOS 96% vs. 74% for *B2Mmut*/*CDKN2Bdel* *p* = 0.03, and 60% for *TP53mut* *p* < 0.0001) (Fig. 3).

**Table 4:** GAs and pathways associated with response and/or survival

Gene	*N*	HR PFS (95% CI)	*p* value	BH *p*	HR OS (95% CI)	*p* value	BH *p*
*CDKN2A*	25	1.4 (0.6, 3.0)	0.391	0.687	1.7 (0.6, 4.4)	0.314	0.847
*KMT2D*	25	1.8 (0.9, 3.8)	0.103	0.428	1.2 (0.4, 3.3)	0.754	0.896
*BCL2*	21	1.9 (0.9, 4.0)	0.114	0.428	0.9 (0.3, 3.1)	0.853	0.896
*B2M*	20	1.4 (0.6, 3.1)	0.483	0.725	2.9 (1.1, 7.6)	**0.027**	**0.204**
*BCL6*	19	0.4 (0.1, 1.4)	0.161	0.482	0.9 (0.3, 3.1)	0.866	0.896
*TP53*	**19**	4.5 (2.1, 9.5)	**<0.0001**	**0.001**	5.8 (2.1, 16.0)	**0.001**	**0.011**
*CREBBP*	18	1.5 (0.7, 3.5)	0.319	0.687	1.9 (0.7, 5.4)	0.225	0.844
*MYD88*	18	1.4 (0.6, 3.3)	0.408	0.687	0.6 (0.1, 2.7)	0.534	0.847
*CDKN2B*	15	2.2 (0.9, 5.0)	0.076	0.428	2.5 (0.9, 7.1)	0.079	0.395
*TNFAIP3*	15	0.7 (0.2, 2.3)	0.569	0.764	1.4 (0.4, 5.0)	0.564	0.847
*EZH2*	14	0.7 (0.2, 2.4)	0.611	0.764	0.4 (0.1, 3.2)	0.411	0.847
*REL*	12	0.5 (0.1, 2.3)	0.412	0.687	0.9 (0.2, 3.9)	0.896	0.896
*PIM1*	11	0.9 (0.3, 2.8)	0.800	0.858	0.4 (0.0, 2.9)	0.348	0.847
*TNFRSF14*	11	1.0 (0.3, 3.4)	0.972	0.972	0.6 (0.1, 4.2)	0.565	0.847
Pathway							
Translation	**10**	**2.3 (0.9, 6.0)**	**0.091**	**0.453**	**4.9 (1.7, 13.8)**	**0.003**	**0.039**
Tumor suppressor/p53	**56**	**3.2 (1.5, 7.0)**	**0.003**	**0.052**	**4.3 (1.4, 13.2)**	**0.011**	**0.081**
Immune evasion	31	1.1 (0.5, 2.3)	0.886	0.961	2.2 (0.9, 5.6)	0.095	0.473
Epigenetic histone all	49	1.9 (0.9, 3.8)	0.083	0.453	1.9 (0.7, 4.9)	0.184	0.689

Differing values with unadjusted *p* < 0.05 depicted in bold

*BH* FDR-adjusted *p* value (Benjamini−Hochberg), *R/R* relapsed refractory, *SNV* short nucleotide variant, *tFL* transformed follicular lymphoma

## Discussion

Clinical trials that select patients for novel targeted therapies based on GAs require large-scale standardized sequencing endeavors, which can be facilitated by targeted DNA and RNA sequencing of FFPE specimens. This work describes the application of a CLIA-compliant integrated DNA and RNA targeted sequencing panel to a retrospective cohort of 198 FFPE DLBCL specimens for the identification of GAs with potential clinical significance.

Prior studies defining the genomic landscape of DLBCL have produced highly variable results^2^. For example, reported frequencies of *CREBBP*/*EP300* mutations in DLBCL have ranged widely from 5 to 44%^3–7^. We detected alterations in the histone acetyltransferases *CREBBP/EP300* in 21% of clinical FFPE DLBCL specimens. This frequency corresponds to the approximate 20% response rates to HDAC inhibitors observed in unselected patients with DLBCL, providing a biologic rationale for a clinical trial using *CREBBP/EP300* mutation as a genomic biomarker to select DLBCL patients for treatment with the HDAC inhibitors (NCT02282358)^19^. Likewise, prior studies reported *EZH2* mutations frequencies as high as 24%^20^, whereas we found *EZH2mut* in 11% of our cohort, a difference that would have major implications for designing a trial with sequencing-based selection of patients for treatment with EZH2 inhibitors.

Comprehensive genomic profiling also detected uncommon GAs (occurring at <3% frequency) with potential therapeutic relevance, which could help identify patients for genetically defined basket trials or select molecularly targeted therapies (supplementary excel file 2). For example, *BRAF* V600E and L597R/V mutations which may predict for response to BRAF or MEK inhibitors^21^. Likewise, we identified mutations in *ID3* (L70P, P98R, Q100P) and *TCF3* (N551K), which had been previously described, predominantly in Burkitt lymphoma, to confer dependence on PI3K pathway signaling, and may indicate a potential therapeutic vulnerability^22^.

In our study, 90% of the patients had at least one potentially targetable GA which could guide selection for clinical trials. Nearly two-thirds of these patients harbored two or more such targets. When using genomic profiling to define genomic biomarkers, it will be important to determine how variant allele frequencies (VAFs) of actionable mutations impact responses to molecularly targeted therapies. For example, we found that gain-of-function mutations in *MYD88* L273P (a.k.a. L265P) exhibited VAFs that ranged from 9 to 74% (supplementary table 3). Thus, if the *MYD88* mutations were targeted with a downstream IRAK1/4 inhibitor, the question arises as to whether tumors showing subclonal GAs with low VAFs will respond similarly to those with a high VAF dominant clone^23^. Although the impact of clonal and subclonal GAs on response to targeted therapies will become clearer as empiric observations are gathered, consensus regarding this question will need to be established soon, in order to design clinical trials that prospectively select patients based on genomic biomarkers^16^.

Consistent with previous reports, using non-clinical genome sequencing assays in the research setting, we confirmed that *KMT2Dmut* to be the most common SNV, present in 26% of de novo cases^3–5,7,24,25^. Similarly, *TP53mut* was noted in 16% of de novo disease and was enriched among R/R patients (33%)^3–5,24,25^. Further, in agreement with previous literature, certain GAs were highly associated with cell of origin designation, highlighting the need to account for molecular case-mix when comparing results from different genomic profiling studies. Compared to non-GCB, GCB samples were enriched for *BCL2trans*, *CREBBPmut*, *KMT2Dmut*, *TNFRSF14mut*, and *EZH2mut*, in keeping with rates described previously in the literature^6,7,20,24^. Likewise, non-GCB samples were enriched for *CD79Bmut, BCL6trans*, *MYD88mut*, *ETV6mut*, and *PRDM1mut* also at similar rates as previously described^6,7,20,24^.

Despite an accumulating body of research into the genomic landscape of DLBCL, very few GAs have been found to be associated with treatment refractoriness or disease relapse. Our study confirms prior associations between *TP53mut* and survival^26,27^. Though marginally significant, *CDKN2A/Bdel* and *B2Mmut* were also found to be associated with shorter OS^28–30^.

As larger sequencing cohorts are assembled, future studies will continue to refine the association between GAs and treatment outcomes. While many large centers use their own home-grown sequencing assays to select patients for targeted therapy, the use of a commercially available clinical assay can facilitate the application of precision medicine at local hospitals and doctor offices, in addition to facilitating the conduct of clinical trials across several institutions. Furthermore, the use of a standardized commercial assay may allow comparing the results of different clinical trials that select patients based on specific genetic alterations. Typically, the cost of a commercial assay is higher than institutional platforms. However, only large institutions are able to develop their own sequencing assays, leaving smaller centers to depend on third-party commercial vendors.

## Electronic supplementary material

Supplementary Material

File 1: Summary of genomic abnormalities

File 2: Potentially targetable genomic alterations by level of evidence
