# Supplementary material for: Integrated DNA/RNA targeted genomic profiling of diffuse large B-cell lymphoma using a clinical assay
Source: Blood Cancer J. 2018 Jun 12;8(6):60. doi: 10.1038/s41408-018-0089-0 (PMC5997645; doi:10.1038/s41408-018-0089-0)
Supplement: Supplementary file 1 — Supplementary Material [file 41408_2018_89_MOESM1_ESM.pdf]

Contents

Supplemental Methods 2

Suppl. table 1: Summary of published validation tests of the FoundationOne pipeline 6

Suppl. table 2: Molecular pathways definition 7

Suppl. table 3: Summary of GAs for the entire cohort by type and allele frequency 8

Suppl. table 4: Summary of key genomic alterations by cell of origin 9

Suppl. table 5: Summary of key involved pathways by cell of origin 10

Suppl. figure 1: Waterfall plot of genomic alterations by type 11

Suppl. figure 2: Co-mutation plot 12

Suppl. figure 3: Genomic alterations by cell of origin 13

Suppl. Excel file: Summary of genomic abnormalities. 14

Suppl. Excel file: Potentially targetable genomic alterations by level of evidence. 14

Supplemental Methods

Description of workflow

DNA and RNA from each patient are extracted and made into barcoded libraries through separate workflow streams. The DNA and cDNA undergo library construction and hybrid selection on independent plates. DNA and RNA samples from the same patient then converge in an analysis pipeline using the plate names and shared specimen ID.

DNA and RNA extraction

DNA and RNA are extracted from FFPE samples as previously described.[1,2] A 5µm FFPE section is stained using hematoxylin and eosin and reviewed by a pathologist to confirm ≥ 20% tumor nuclei and a tissue volume of ≥2mm^3. A macro-dissection of samples is performed when warranted in order to enrich for tumor content. DNA and RNA are each extracted from 40 µm (typically 4x10µm) of unstained FFPE sections.

DNA extraction: FFPE samples are deparaffinized and then digested with a proteinase K buffer for 12–24 h followed by purification with the Promega Maxwell 16 Tissue LEV DNA kit. Double-stranded DNA is quantified by a Picogreen fluorescence assay using the provided lambda DNA standards (Invitrogen). 50–200 ng of dsDNA in 50–100 µl water in microTUBEs is fragmented to ~200 bp by sonication (3 min, 10% duty, intensity = 5, 200 cycles/burst; Covaris E210) before purification using a 1.8× volume of AMPure XP Beads (Agencourt). Samples yielding <50 ng of extracted DNA are considered failed (estimated failure rate 4.9%[1]).

RNA extraction: FFPE samples are deparaffinized and then digested with proteinase K lysis buffer at 56ºC for 15min followed by 80ºC for 15min. The lysate is treated with freshly prepared DNase at room temperature for 10 min and then purified using the Promega Maxwell CSC RNA FFPE kit. Samples are quantified for RNA yield using RiboGreen (LifeTech). Samples with RNA yield ≥3.5ng/µL proceed to cDNA synthesis. RNA is normalized 500ng in a volume of 22.7µL. A cDNA primer mixture of random hexamer (IDT) and oligo dT (IDT) are annealed to the template RNA at 65ºC for 5min. First strand synthesis is performed using M-MLV RT RNase(H-) (Promega #M3683) 25°C 10 min, 40°C 50 min, 85°C 5 min. Second strand synthesis follows using the NEB Second strand mRNA synthesis kit (#E6111L) and incubated at 16ºC for 30min. The entire cDNA product is sheared by sonication to ~200bp fragment size (3 min, 10% duty, intensity = 5, 200 cycles/burst; Covaris E210) before purification using 1.8x SPRI clean up.

Library construction and hybrid selection

Solution hybridization is performed using pools of 5′ biotinylated 120 bp oligonucleotide DNA baits (Integrated DNA Technology); a pool of 35,845 baits for the DNA libraries and pool of 22,656 baits for the cDNA libraries. Baits were designed by taking overlapping 120 bp DNA sequence intervals covering target exons (60 bp overlap) and introns (20 bp overlap), with a minimum of three baits per target; SNP targets were allocated one bait each. Intronic baits were filtered for repetitive elements as defined by the UCSC Genome RepeatMasker track.[3] Hybrid selection of targets demonstrating reproducibly low coverage was boosted by increasing the number of baits for these targets.

SPRI purification and subsequent library construction with the NEBNext kits (E6040S, NEB), containing mixes for end repair, dA addition and ligation, are performed in 96-well plates (Eppendorf) on a Bravo Benchbot (Agilent) using the “with-bead” protocol[4] to maximize reproducibility and library yield. 500–2,000 ng of sequencing library is and suspended in water, heat denatured at 95 °C for 5 min and then incubated at 68 °C for 5 min before addition of the baitset reagent and Cot, salmon sperm and adaptor-specific blocker DNA in hybridization buffer. After a 24-h incubation, the library-bait duplexes are captured on paramagnetic MyOne streptavidin beads (Invitrogen) and off-target library is removed by washing once with 1× SSC at 25 °C and four times with 0.25× SSC at 55 °C. The PCR master mix is added to directly amplify (12 cycles) the captured library from the washed beads.[4] After amplification, the samples are 1.8× SPRI purified, quantified by qPCR (Kapa) and sized on a LabChip GX (Caliper). Samples yielding <500 ng of sequencing library, or with a mean insert size >400 bp, are considered failed. Size selection was not done. Libraries are normalized to 1.05 nM and pooled such that each Illumina HiSeq 2500 lane has up to four samples each (32 per flowcell), before 49 × 49 paired-end sequencing using manufacturer's protocols to ~500× unique coverage for DNA and to >3 M unique on-target pairs for cDNA.

Sequence data processing

Sequence data were mapped to the human genome (hg19) using BWA aligner v0.5.9.[5] PCR duplicate read removal and sequence metric collection was done using Picard 1.47 (http://picard.sourceforge.net/) and Samtools 0.1.12a33. Local alignment optimization was performed using GATK 1.0.4705.[6] Variant calling was done only in genomic regions targeted by the test.

Base substitutions, indels, and copy number analysis

Samples with median exon coverage in the range 150 to 250× are considered qualified, whereas those with coverage <150× are considered failed. Significant non-synonomous variants were defined as any somatic alteration annotated in the COSMIC database (v62), as well as clear inactivating mutations (i.e. truncations or deletions) in established tumor suppressor genes.[7] For base substitutions, the mutant allele frequency (MAF) cutoff used was 1% for known somatic variants (based on COSMIC v62) and 5% for novel somatic variants.

To detect indels, *de novo* local assembly in each targeted exon was performed using the de Bruijn approach.[8] Key steps are:

1. Collecting all read-pairs for which at least one read maps to the target region.
2. Decomposing each read into constituent *k*-mers and constructing an enumerable graph representation (de Bruijn) of all candidate nonreference haplotypes present.
3. Evaluating the support of each alternate haplotype with respect to the raw read data to generate mutational candidates. All reads are compared to each of the candidate haplotypes through ungapped alignment, and a 'vote' for each read is assigned to the candidate with best match. Ties between candidates are resolved by splitting the read vote, weighted by the number of reads already supporting each haplotype. This process is iterated until a 'winning' haplotype is selected.

4. Aligning candidates against the reference genome to report mutation calls. Indel candidates arising from direct read alignment were also considered.

For indels, the MAF cutoff was 3% for known somatic variants and 10% for novel somatic variants. Additional details were described previously.[1]

CNA detection was achieved using a comparative genomic hybridization (CGH)-like method. A log-ratio profile of the sample is obtained by normalizing the sequence coverage at all exons against a process-matched normal control. This profile was corrected for GC-bias, segmented and interpreted using allele frequencies of sequenced SNPs to estimate tumor purity and copy number at each segment as previously described.[1] . Fitting was performed using Gibbs sampling, assigning absolute copy number to all segments. Model quality was reviewed and alternative explanations considered,[9] and focal amplifications are called at segments with ≥6 copies (or ≥7 for triploid; ≥8 for tetraploid tumors) and homozygous deletions at 0 copies, in samples with purity >20%.

Rearrangement calling methods

Gene rearrangements were detected by identifying clusters of chimeric read pairs from both DNA (pairs mapping >10 kbp apart or on different chromosomes) and RNA (pairs mapping to refSeq sequences corresponding to different genes or to genomic loci >10 kbp apart, and reads with suboptimal mapping were aligned to whole genome references). Alignments to the 2 different references were then merged and calibrated based on the full genome reference (hg19) for fusion detection. Chimera clusters were filtered for repetitive sequence (average mapq >30) and by distribution of mapped positions (SD >10). Identified rearrangements were then annotated according to the genomic loci of both clusters and categorized as gene fusions (eg, BCR-ABL1), gene rearrangements (eg, IGH-BCL2), or truncating events (eg, TP53 rearrangement). Rearrangement candidates were then filtered based on number of chimera reads supporting the rearrangement events (for documented fusions, a minimum 10 chimera reads are required; for putative somatic driver rearrangements, 50 chimera reads are required).

In addition to the de novo rearrangement detection method described above, reads were also separately aligned to a custom reference library generated based on common fusions and rearrangements. Fusions were detected based on the observation of reads aligned across the junction of rearrangement breakpoints. Immunoglobulin heavy locus (IGH) rearrangements were detected by targeting rearrangement hotspots of both common immunoglobulin fusion partner genes (major and minor translocations involving MYC, BCL2, and CCND1), as well as IGH breakpoint regions.

References

1. Frampton GM, Fichtenholtz A, Otto GA, et al. Development and validation of a clinical cancer genomic profiling test based on massively parallel DNA sequencing. *Nat Biotechnol*. 2013;31(11):1023-1031.

2. He J, Abdel-Wahab O, Nahas MK, et al. Integrated genomic DNA/RNA profiling of hematologic malignancies in the clinical setting. *Blood*. 2016;127(24).

3. Karolchik D, Hinrichs AS, Furey TS, et al. The UCSC Table Browser data retrieval tool. *Nucleic Acids Res*. 2004;32(90001):493D-496.

4. Fisher S, Barry A, Abreu J, et al. A scalable, fully automated process for construction of sequence-ready human exome targeted capture libraries. *Genome Biol*. 2011;12(1):R1.

5. Li H, Durbin R. Fast and accurate long-read alignment with Burrows-Wheeler transform. *Bioinformatics*. 2010;26(5):589-595.

6. DePristo MA, Banks E, Poplin R, et al. A framework for variation discovery and genotyping using next-generation DNA sequencing data. *Nat Genet*. 2011;43(5):491-498.

7. Forbes SA, Beare D, Gunasekaran P, et al. COSMIC: exploring the world's knowledge of somatic mutations in human cancer. *Nucleic Acids Res*. 2015;43(D1):D805-D811.

8. Compeau PEC, Pevzner PA, Tesler G. How to apply de Bruijn graphs to genome assembly. *Nat Biotechnol*. 2011;29(11):987-991.

9. Van Loo P, Nordgard SH, Lingjaerde OC, et al. Allele-specific copy number analysis of tumors. *Proc Natl Acad Sci*. 2010;107(39):16910-16915.

Suppl. table 1: Summary of published validation tests of the FoundationOne pipeline

**Cell lines - concordance with known abnormalities at various dilutions/coverage**		
SNVs	2 pools of 10 normal cell lines (total 2,057 SNVs at variable VAFs)<br>Several dilutions to simulate lower coverages	At median exon coverage of 600x-700x: >99% Sensitivity and >99% PPV even at VAF<5%<br>At median exon coverage of 250x: >98% sensitivity and >99% PPV even at VAF<5%<br>Significant reduction of sensitivity was observed at VAF<10% & coverage < 100x.
Indels	28 cell lines with 47 somatic indels.<br>41 pools of 2-10 cell lines.	At median exon coverage of 670x: 98% sensitivity at VAF ≥20%; 97% at VAF ≥10%; and 88% at VAF ≥5%; PPV > 99%<br>At median exon coverage of 250x: 98% sensitivity at VAF ≥20%; 92% at VAF ≥10%.
CNAs	Pooled 7 cell lines with 19 focal amplifications (6-15 copies in 15 genes) and 9 homozygous deletions (6 genes) with matched normal cells at sequential mixtures of 10-75%	Sensitivity and PPV 99% for deletion and CNA>7 copies at tumor purity ≥30%; Overall sensitivity >80% for CAN<7 and tumor content 20-30%.
Rearrangements	21 cell lines with 28 known rearrangements mixed with pooled normal cell lines at sequential mixtures of 10-50%	DNAseq - 100% sensitivity for fusion detection (161/161) above 20% tumor fraction.<br>PPV >98% (245/248) with 3 false positive at marginal readings.<br>RNAseq - 100% sensitivity for fusion detection (161/161) above 25% tumor fraction.
**Concordance with alternative methods**		
Mass spectrometry<br>(iPlex - Sequenom/Agena)	113 specimens 91 mutations (solid malignancies)<br>76 specimens 113 genes (hematologic malignancies)	97% concordance for solid tumors.<br>99% concordance for hematologic tumors.
Other NGS<br>(AmpliSeq - Thermo Fisher)	21 samples with low frequency calls (<10% VAF)	95% concordance (20/21)
PCR - Gene rearrangement	12 MLL-PTD<br>14 controls	97% accuracy 11/12 MLL-PTD<br>14/14 negative controls. The 1 missed case d/t present MLL-PTD but below calling threshold.
FISH - IgH rearrangement and known cell lines	10 cell-lines<br>38 FISH	94% concordance<br>2 FP; 1 FN
FISH - CNAs	119 specimens with CNAs (solid tumors)	98% accuracy (1 FP; 1 FN)
**Assay reproducibility and comparison of FFPE to fresh specimen**		
Assay reproducibility	13 samples X 5 replicates	97% concordance with missed case d/t present alterations below calling threshold.
Long-term reproducibility	1 pooled RNA sample<br>1 pooled DNA sample<br>Sequenced repeatedly (134) over 5 months.	100% concordance for 303 SNVs<br>100% concordance for rearrangement by RNA.
Comparison of FFPE to blood	5 matched normal blood FFPE samples (13 germline alterations)	100% Sensitivity though many SNVs highlighted only on the FFPE sample all at VAF < 5%.

Reproduced from Frampton GM, Fichtenholtz A, Otto GA, et al. Development and validation of a clinical cancer genomic profiling test based on massively parallel DNA sequencing. Nat Biotechnol. 2013;31(11):1023-1031. and from He J, Abdel-Wahab O, Nahas MK, et al. Integrated genomic DNA/RNA profiling of hematologic malignancies in the clinical setting. *Blood*. 2016;127(24).

Suppl. table 2: Molecular pathways definition

**Pathway**	**Involved gene**
Histone epigenetic	*ASXL1, BCOR, BRD4, CREBBP, ELP2, EP300, EZH2, HDAC7, HIST1H1C, HIST1H1E, HIST1H2BJ*
DNA epigenetic	*DNMT3A, IDH2, TET2*
SWI SNF epigenetic	*ARID1A, ARID2, PRBM1, SMARCA4*
Epigenetic cofactors	*ASXL1, BCOR, BRD4, NCOR1, NCOR2, TBL1XR1, TRRAP, ZMYM3*
BCR NFKB	*BCL10, BIRC3, CARD11, CD79B, MALT1, MAP3K14, MYD88, REL, TNFAIP3, TNFRSF11A, TNFRSF14*
JAK STAT	*ELP2, JAK1, JAK2, PIM1, SOCS1, TYK2*
RAS MAPK	*BRAF, CIC, EGFR, ERBB2, ERBB3, FLT1, FLT3, FLT4, FRS2, KRAS, MAP2K1*
PI3K AKT TOR	*AKT1, AXL, FLCN, FRS2, HGF, IGF2, PIK3CA, PIK3C2G, PIK3C3, PIK3R1, PTEN*
NOTCH MYC	*APH1A, FBXW7, MYC, MIB1, NCSTN, NOTCH1, NOTCH2, NOTCH4, SPEN*
WNT	*APC, AXIN1, FZD8, WNT10B*
Transcription factors	*BCL6, CDC73, CIC, CUX1, ERG, ETS1, ETV6, FBXO11, FOXO1, ID3, IKZF1*
Tumor suppressor	*APC, ATM, AXIN1, BACH1, BAP1, BRCA2, BRIP1, CDKN2A, CDKN2B, FANCG, FANCL, TP53*
DNA Damage	*ATM, BACH1, BAP1, BRCA2, BRIP1, CHEK1, FANCG, FANCL, FBXO31, MDM2, MDM4*
Cell Cycle	*BRSK1, BTG1, BTG2, CCND1, CCND3, CCNE1, CDK6, CDKN1B, CDKN2C, CHEK1, CKS1B*
RNA processing	*CDK12, CIRBP, DDX3X, DIS3, PUS10, SF3B1, SRSF2, USAF1, XPO1, ZRSR2*
Cell death	*BCL2, CASP8, DEDD2, FAS, MCL1*
Immune evasion	*B2M, CD58, CD70, CD274, CIITA, HLA-A, IL4R, MARCH1, PDCD1LG2, TRG*
Metabolism	*CD36, GRIN2A, IDH2, LRP1B, LRRK2, OSBPL10, PC, SDHA, SDHC, SDHD, SEC22B*
Translation	*CIRBP, EIF4A2, HSP90AA1, HSP90AB1, MYC, SBDS*
Transcription	*CDC73, ELP2, MED12, TAF1*
GPCR	*CXCR4, GNA13*
Phosphatase	*PPP2R1A, PTPN2, PTPN6, SETBP1*
Adhesion cytoskeleton	*ARHGAP24, ARHGAP26, ARHGAP27, CDH1, DDR2, DSCAML1, LPP, RELN, RHOA, SLAMF1, STK11*

Suppl. table 3: Summary of GAs for the entire cohort by type and allele frequency

Gene	Total alt.	SNVs	Allele frequency	Trans	Amp	Del	Total alterations UKS	SNVs UKS	Allele frequency UKS	Trans UKS	Amp UKS	Del UKS
IGH	76	0	NA	76	0	0	1	0	NA	0	0	1
KMT2D	62	59	0.33 (0.07, 0.74)	3	0	0	24	24	0.44 (0.06, 0.66)	0	0	0
CDKN2A	54	13	0.27 (0.02, 0.92)	2	0	40	6	5	0.49 (0.06, 0.52)	1	0	0
TP53	48	48	0.50 (0.02, 0.92)	0	0	0	2	2	0.12 (0.11, 0.14)	0	0	0
BCL2	46	22	0.40 (0.04, 0.88)	45	0	0	32	27	0.36 (0.05, 0.55)	5	0	0
BCL6	37	2	0.56 (0.21, 0.91)	35	0	0	16	14	0.36 (0.08, 0.60)	0	0	2
MYD88	36	36	0.36 (0.09, 0.74)	0	0	0	6	4	0.38 (0.36, 0.50)	2	0	0
CREBBP	35	32	0.36 (0.15, 0.75)	3	0	0	25	25	0.43 (0.16, 0.94)	0	0	0
B2M	33	32	0.44 (0.05, 0.88)	1	0	1	13	12	0.28 (0.06, 0.78)	1	0	0
CDKN2B	32	0	NA	2	0	30	3	2	0.36 (0.24, 0.48)	1	0	0
TNFAIP3	24	21	0.30 (0.06, 0.78)	0	0	3	8	8	0.42 (0.06, 0.57)	0	0	0
EZH2	21	21	0.33 (0.02, 0.56)	0	0	0	4	4	0.48 (0.42, 0.52)	0	0	0
PIM1	20	20	0.40 (0.03, 0.88)	0	0	0	40	40	0.30 (0.06, 0.84)	1	0	0
TNFRSF14	20	15	0.54 (0.05, 0.91)	1	0	4	16	16	0.42 (0.12, 0.93)	0	0	0
CARD11	19	18	0.24 (0.03, 0.51)	1	0	0	16	16	0.32 (0.14, 0.68)	0	0	0
ARID1A	16	16	0.34 (0.15, 0.44)	0	0	0	16	16	0.46 (0.05, 0.53)	0	0	0
REL	16	0	NA	0	16	0	8	6	0.18 (0.07, 0.86)	0	0	2
CD79B	15	15	0.37 (0.16, 0.84)	0	0	0	20	20	0.26 (0.08, 0.83)	0	0	0
FAS	15	15	0.31 (0.07, 0.67)	0	0	0	11	11	0.24 (0.06, 0.50)	0	0	0
MYC	15	0	NA	13	2	0	12	12	0.38 (0.07, 0.61)	0	0	0
BCL7A	14	14	0.32 (0.13, 0.54)	0	0	0	22	22	0.34 (0.07, 0.60)	0	0	0
BCL10	12	11	0.18 (0.03, 0.49)	1	0	0	3	2	0.09 (0.07, 0.11)	1	0	0
CD58	12	8	0.28 (0.10, 0.39)	2	0	2	8	8	0.24 (0.11, 0.37)	0	0	0
CD70	11	9	0.56 (0.30, 0.86)	0	0	2	12	12	0.33 (0.11, 0.48)	0	0	0
ETV6	11	9	0.24 (0.08, 0.83)	2	0	0	9	9	0.34 (0.10, 0.79)	0	0	0
NOTCH2	11	11	0.36 (0.08, 0.74)	1	0	0	18	16	0.50 (0.06, 0.59)	2	0	0
PRDM1	11	9	0.33 (0.11, 0.72)	1	0	1	7	7	0.46 (0.08, 0.81)	0	0	0
TET2	10	10	0.28 (0.10, 0.56)	0	0	0	3	3	0.43 (0.15, 0.47)	0	0	0

SNV- short nucleotid variant; UKS – alterations of unknown significance. Genomic abnormalities occurring in ≥5% of the cohort.
Variant actionability was graded based on the OncoKB guideline. Level 1 was defined as alterations recognized by the FDA as predictive of response to an approved drug in DLBCL. Level 2 included non-FDA predictive biomarkers for response in DLBCL (2A) or FDA approved biomarkers for response in a different malignancy (2B). Level 3 includes alterations supported by compelling data from clinical trials in DLBCL (3A) or another malignancy (3B). Level 4 are candidate biomarkers for response based on early clinical or preclinical studies.

Suppl. table 4: Summary of key genomic alterations by cell of origin

	[ALL]	GCB	non-GCB	NA	p value*	BH p*
	198	95	82	21		
SNVs	191 (96.5)	91 (95.8)	81 (98.8)	19 (90.5)	0.375	
SNVs per/pt.	**4 (0, 9)**	**4 (0, 9)**	**3 (0, 9)**	**4 (0, 7)**	**0.019**	
SNVs of UKS per/pt.	15.5 (3, 45)	15 (3, 45)	15.5 (4, 40)	18 (10, 38)	0.931	
Amplifications	36 (18.2)	19 (20.0)	14 (17.1)	3 (14.3)	0.760	
Deletions	57 (28.8)	26 (27.4)	25 (30.5)	6 (28.6)	0.771	
**Translocations**	**112 (56.6)**	**62 (65.3)**	**37 (45.1)**	**13 (61.9)**	**0.011**	
**Total number of GAs**	**6 (0, 13)**	**6 (0, 13)**	**5 (0, 13)**	**7 (0, 10)**	**0.028**	
**KMT2D**	**62 (31.3%)**	**41 (43.2%)**	**17 (20.7%)**	**4 (19.0%)**	**0.003**	**0.015**
CDKN2A	54 (27.3%)	23 (24.2%)	26 (31.7%)	5 (23.8%)	0.346	0.538
TP53	48 (24.2%)	27 (28.4%)	16 (19.5%)	5 (23.8%)	0.229	0.401
**BCL2**	**46 (23.2%)**	**38 (40.0%)**	**4 (4.88%)**	**4 (19.0%)**	**<0.001**	**0.000**
**BCL6**	**37 (18.7%)**	**10 (10.5%)**	**22 (26.8%)**	**5 (23.8%)**	**0.009**	**0.039**
**MYD88**	**36 (18.2%)**	**12 (12.6%)**	**23 (28.0%)**	**1 (4.76%)**	**0.017**	**0.061**
**CREBBP**	**35 (17.7%)**	**26 (27.4%)**	**5 (6.10%)**	**4 (19.0%)**	**<0.001**	**0.004**
B2M	33 (16.7%)	16 (16.8%)	14 (17.1%)	3 (14.3%)	1.000	1.000
CDKN2B	32 (16.2%)	15 (15.8%)	14 (17.1%)	3 (14.3%)	0.979	1.000
TNFAIP3	24 (12.1%)	7 (7.37%)	12 (14.6%)	5 (23.8%)	0.189	0.353
**EZH2**	**21 (10.6%)**	**16 (16.8%)**	**4 (4.88%)**	**1 (4.76%)**	**0.023**	**0.072**
PIM1	20 (10.1%)	7 (7.37%)	10 (12.2%)	3 (14.3%)	0.406	0.568
**TNFRSF14**	**20 (10.1%)**	**16 (16.8%)**	**3 (3.66%)**	**1 (4.76%)**	**0.010**	**0.039**
CARD11	19 (9.60%)	9 (9.47%)	8 (9.76%)	2 (9.52%)	1.000	1.000
ARID1A	16 (8.08%)	6 (6.32%)	7 (8.54%)	3 (14.3%)	0.783	0.934
REL	16 (8.08%)	11 (11.6%)	5 (6.10%)	0 (0.00%)	0.315	0.518
**CD79B**	**15 (7.58%)**	**0 (0.00%)**	**13 (15.9%)**	**2 (9.52%)**	**<0.001**	**0.003**
FAS	15 (7.58%)	4 (4.21%)	9 (11.0%)	2 (9.52%)	0.152	0.305
MYC	15 (7.58%)	10 (10.5%)	2 (2.44%)	3 (14.3%)	0.067	0.143
BCL7A	14 (7.07%)	10 (10.5%)	2 (2.44%)	2 (9.52%)	0.067	0.143
BCL10	12 (6.06%)	5 (5.26%)	6 (7.32%)	1 (4.76%)	0.801	0.934
CD58	12 (6.06%)	5 (5.26%)	5 (6.10%)	2 (9.52%)	1.000	1.000
CD70	11 (5.56%)	5 (5.26%)	6 (7.32%)	0 (0.00%)	0.801	0.934
**ETV6**	**11 (5.56%)**	**2 (2.11%)**	**9 (11.0%)**	**0 (0.00%)**	**0.034**	**0.085**
NOTCH2	11 (5.56%)	4 (4.21%)	7 (8.54%)	0 (0.00%)	0.381	0.561
**PRDM1**	**11 (5.56%)**	**2 (2.11%)**	**9 (11.0%)**	**0 (0.00%)**	**0.034**	**0.085**
TET2	10 (5.05%)	4 (4.21%)	6 (7.32%)	0 (0.00%)	0.517	0.689

* Unadjusted and BH adjusted p values reflect the comparison of GCB to non-GCB disease (i.e. excludes Unclassified).

BH – FDR adjusted p value (Benjamini-Hochberg); GCB – germinal center; NA – not available; SNV- short nucleotid variant; UKS – alterations of unknown significance (per Cosmic v62).

Genomic abnormalities occurring in ≥5% of the cohort. For certain genes alteration may include SNVs, rearrangements and/or CNAs (see Suppl. figure 1). In bold – differing values with unadjusted $p < 0.05$.

Suppl. table 5: Summary of key involved pathways by cell of origin

	[ALL]	GCB	non-GCB	NA	p value*	BH p*
	198	95	82	21		
**Tumor suppressor**	123 (62.1)	63 (66.3)	48 (58.5)	12 (57.1)	0.362	0.597
**Histone epigenetic**	107 (54.0)	61 (64.2)	35 (42.7)	11 (52.4)	0.007	0.063
BCR NFKB	107 (54.0)	47 (49.5)	50 (61.0)	10 (47.6)	0.167	0.397
**Transcription factors**	74 (37.4)	27 (28.4)	38 (46.3)	9 (42.9)	0.021	0.132
**Cell death**	68 (34.3)	46 (48.4)	16 (19.5)	6 (28.6)	0.000	0.002
Immune evasion	53 (26.8)	23 (24.2)	25 (30.5)	5 (23.8)	0.443	0.601
NOTCH MYC	45 (22.7)	21 (22.1)	18 (22.0)	6 (28.6)	1.000	1.000
JAK STAT	35 (17.7)	12 (12.6)	18 (22.0)	5 (23.8)	0.148	0.397
RAS MAPK	35 (17.7)	14 (14.7)	17 (20.7)	4 (19.0)	0.396	0.597
Metabolism	32 (16.2)	21 (22.1)	9 (11.0)	2 (9.5)	0.077	0.367
Cell Cycle	24 (12.1)	15 (15.8)	6 (7.3)	3 (14.3)	0.132	0.397
Translation	22 (11.1)	12 (12.6)	6 (7.3)	4 (19.0)	0.359	0.597
SWI SNF epigenetic	21 (10.6)	10 (10.5)	7 (8.5)	4 (19.0)	0.848	0.597
DNA Damage	21 (10.6)	8 (8.4)	11 (13.4)	2 (9.5)	0.408	1.000
RNA processing	20 (10.1)	12 (12.6)	4 (4.9)	4 (19.0)	0.126	0.397
Epigenetic cofactors	19 (9.6)	10 (10.5)	6 (7.3)	3 (14.3)	0.631	0.800
PI3K AKT TOR	17 (8.6)	11 (11.6)	5 (6.1)	1 (4.8)	0.315	0.597
DNA epigenetic	13 (6.6)	7 (7.4)	6 (7.3)	0 (0.0)	1.000	1.000
Adhesion cytoskeleton	12 (6.1)	6 (6.3)	6 (7.3)	0 (0.0)	1.000	1.000

* p values reflect the comparison of GCB to non-GCB disease (i.e. excludes NA).

GCB – germinal center; NA – not available; SNV- short nucleotid variant; UKS – alterations of unknown significance (per Cosmic v62). Genomic abnormalities occurring in ≥5% of the cohort. For certain genes alteration may include SNVs, rearrangements and/or CNAs (see Suppl. figure 1). In bold – differing values with unadjusted $p < 0.05$.

Suppl. figure 1: Waterfall plot of genomic alterations by type

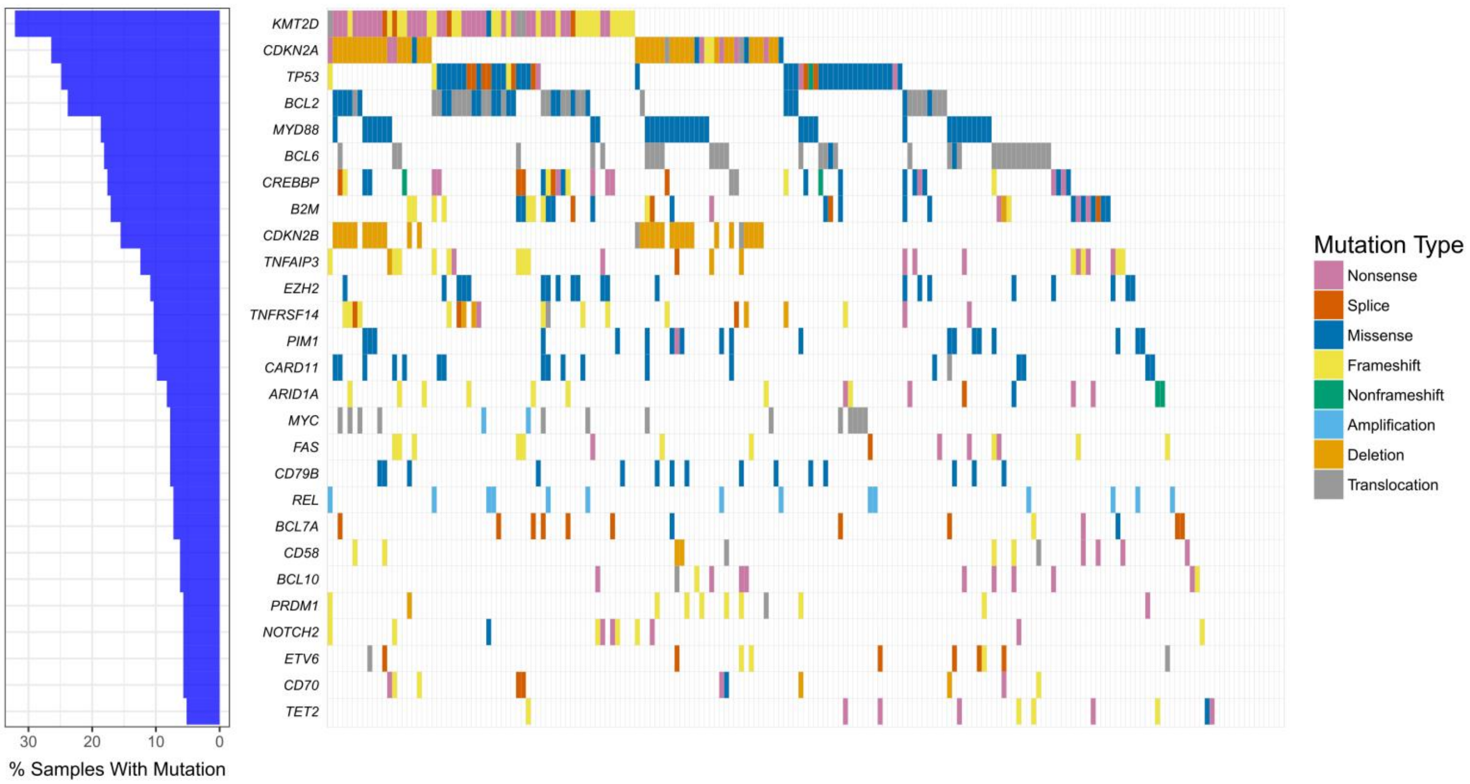

Displaying GA present in 5% or more of patients.

Suppl. figure 2: Co-mutation plot

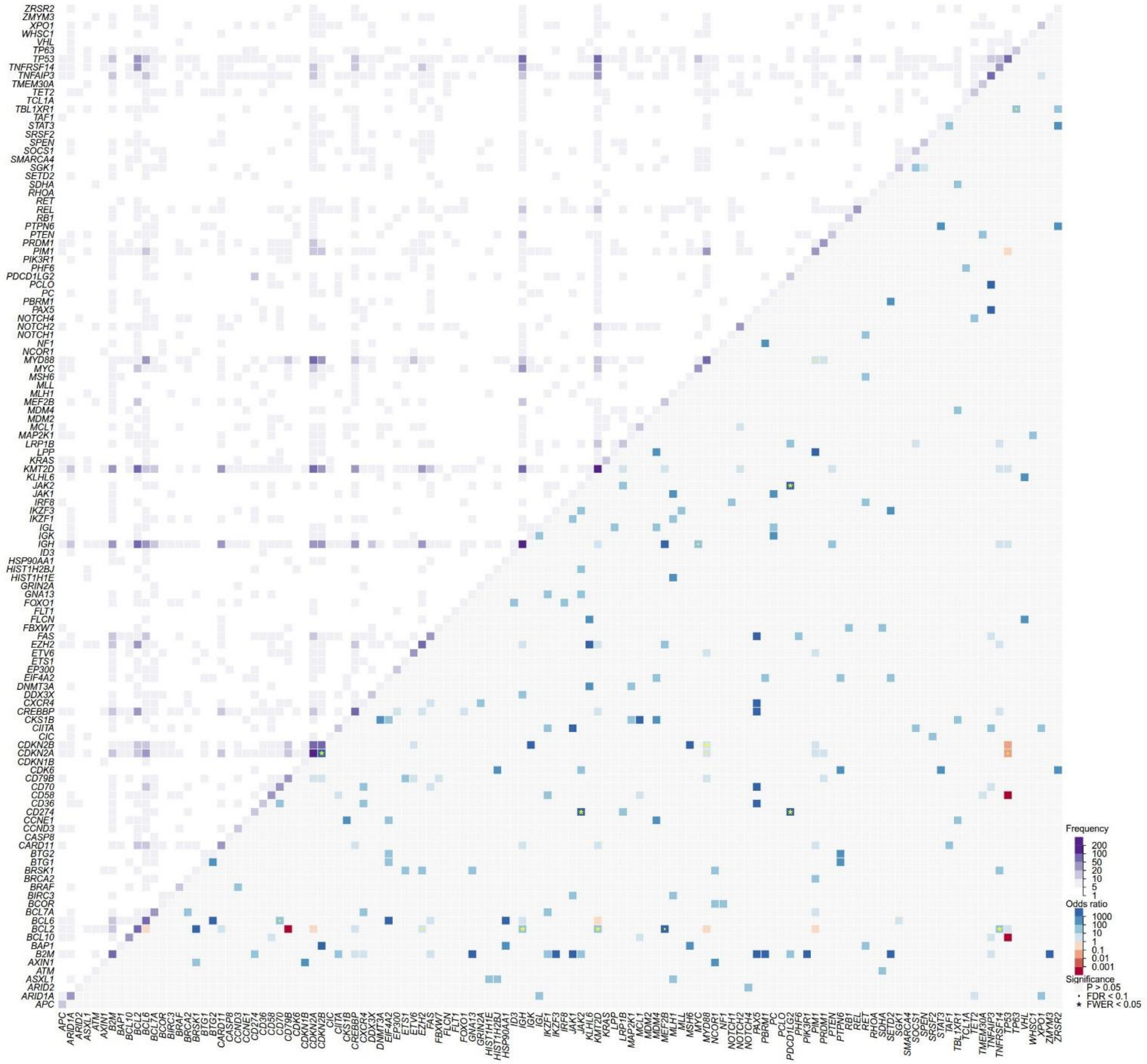

Pairwise analysis of genes for co-occurrence (blue) and anti-co-occurrence plot (red) (lower right triangle). Pairwise frequency calculated as the rate of abnormality B among patients with abnormality A (top right triangle).

Suppl. figure 3: Genomic alterations by cell of origin

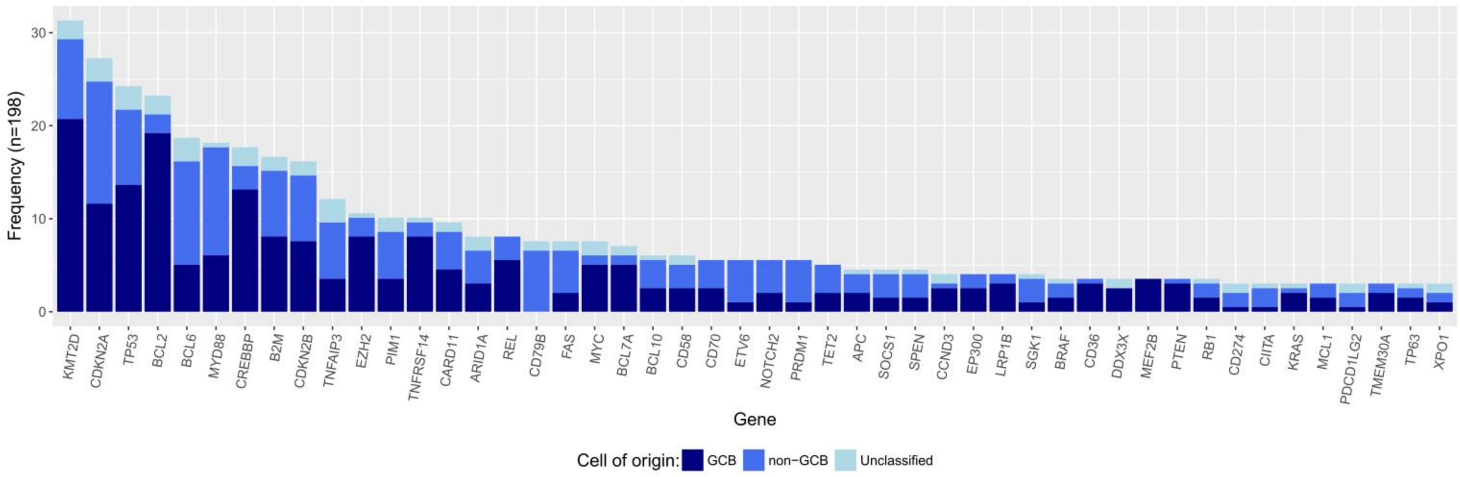

Suppl. Excel file: Summary of genomic abnormalities.

Suppl. Excel file: Potentially targetable genomic alterations by level of evidence.
